# Modelling Tauopathies in *Drosophila*: Insights from the Fruit Fly

**DOI:** 10.4061/2011/598157

**Published:** 2011-12-29

**Authors:** Catherine M. Cowan, Megan A. Sealey, Shmma Quraishe, Marie-Therese Targett, Kristen Marcellus, Douglas Allan, Amritpal Mudher

**Affiliations:** ^1^Centre for Biological Sciences, University of Southampton, University Road, Southampton SO17 3JD, UK; ^2^Department of Cellular and Physiological Sciences, University of British Columbia, Room 2420 Life Sciences Centre, 2350 Health Sciences Mall, Vancouver, BC, Canada V6T 1Z3

## Abstract

*Drosophila melanogaster* is an experimentally tractable model organism that has been used successfully to model aspects of many human neurodegenerative diseases. *Drosophila* models of tauopathy have provided valuable insights into tau-mediated mechanisms of neuronal dysfunction and death. Here we review the findings from *Drosophila* models of tauopathy reported over the past ten years and discuss how they have furthered our understanding of the pathogenesis of tauopathies. We also discuss the multitude of technical advantages that *Drosophila* offers, which make it highly attractive as a model for such studies.

## 1. Tauopathies

Tauopathies are a group of neurodegenerative diseases characterised by abnormally hyperphosphorylated and insoluble aggregates of the microtubule-associated protein tau [1]. They include diseases where the tau pathology is the only neuropathological hallmark (such as frontotemporal dementia, Pick's disease, corticobasal degeneration, progressive supranuclear palsy, and others), as well as diseases where the tau pathology coexists with another pathology (such as Alzheimer's disease (AD), Parkinson's disease, and Creutzfeldt-Jakob disease amongst others). The nature of the tau aggregates, their constituent tau isoforms, and the brain region they deposit in varies depending on the disease. The tau aggregates are found in neuronal as well as glial cells, and the brain regions affected include hippocampal/entorhinal regions, cortical regions, and mid/hind brain regions. Thus, the clinical symptoms range from cognitive impairments to locomotor disabilities [2].

Despite the varied neuropathological and clinical profiles, all tauopathies are characterised by the same tau aberrations: abnormal and hyperphosphorylation [3], misfolding [4], and aggregation [5]. Mutations in the tau gene, which give rise to frontotemporal dementia with Parkinsonism linked to chromosome 17 (FTDP-17) [6], are capable of inducing these tau modifications. Since they are associated with degeneration and dementia, these changes in tau are likely to be responsible for these pathologies. However, the processes that trigger tau abnormalities in sporadic tauopathies have not yet been identified. Moreover, despite studies to investigate the physiological consequences of these tau aberrations in various models of tauopathy, their pathological significance is still debated. The emerging realisation that tau has additional functions in the neuron, other than microtubule stabilisation (reviewed in Morris et al., 2011 [7]), implies that tau abnormalities are likely to impact upon more than one neuronal process. Thus, there may be multiple mechanisms mediating tau toxicity in tauopathies. In addition to dissecting mechanisms of tau toxicity, research efforts are also focused on further understanding of other aspects of tau biology, including its turnover, its regulation by myriad posttranslational modifications, (other than phosphorylation), and its interaction with other disease-associated proteins like amyloid beta (A*β*).

Numerous models of tauopathy, in both vertebrates [8, 9] and invertebrates [10, 11], have been generated to address links between tau biology and pathology. *Drosophila* occupies a unique position amongst model organisms because of the rich history of its use as a genetic model and hence its powerful genetic tractability. In this paper, we first describe some of the attributes of *Drosophila* that make it an excellent choice of organisms to study tauopathies, to test hypotheses about pathogenesis, identify disease mechanisms, and even screen disease-modifying drugs. Then we go on to review important insights about tauopathies that have come from *Drosophila* models over the past ten years.

### 1.1. *Drosophila* as a Model Organism

Aside from the very obvious advantages afforded by its small size, inexpensive maintenance, rapid propagation, and short life span, *Drosophila melanogaster* has a multitude of technical advantages. It is not the intention of this paper to discuss these in any great detail, but merely to highlight the reasons why *Drosophila* may be particularly attractive for modelling aspects of human diseases such as tauopathies and how its attributes can enable further insights to be gained into disease mechanisms.

#### 1.1.1. Insights from *Drosophila* Are Relevant to Humans

*Drosophila* has played a pivotal role in deciphering numerous fundamental biological processes ranging from our understanding of genetics to our current knowledge of important physiological processes including embryogenesis, cell signalling, aging, and circadian rhythms, to name just a few (for a more comprehensive review of the rich history of the use of *Drosophila* as a model in modern biology see Pandey and Nichols 2011 [12]). The fact that the underlying cell/molecular bases for these fundamental biological events are essentially the same from *Drosophila* to mammals highlights the striking conservation of basic physiological processes across the species. This is further underlined by the finding, upon the comparison of the completed human and fly genomes, that over 75% of genes implicated in human disease, have *Drosophila* orthologues [13]. This opens up the exciting possibility that just as the simplicity of *Drosophila* was exploited to gain insight into important mammalian biological processes, we may now use *Drosophila* models to shed light on mechanisms underpinning mammalian diseases. Indeed, aspects of many common human diseases, including neurodegenerative diseases (such as Alzheimer's, Huntington's, Parkinson's—reviewed in Marsh and Thompson, 2006 [14] and Iijima-Ando and Iijima, 2010 [15]), seizure disorders, and affective disorders (such as alcohol addiction [16] and cancers [17]) have been successfully modelled in *Drosophila*. Furthermore, there is scope for the development of additional *Drosophila* models of human disease including cardiovascular disease, inflammatory disease and diabetes (reviewed in Pandey and Nichols 2011 [12]).

#### 1.1.2. *Drosophila* Is Highly Genetically Tractable

100 years of extensive use as an experimental genetic model (as described above), as well as a sequenced and highly annotated genome, provides *Drosophila* researchers with many elegant and powerful genetic tools (see Table 1 and for a comprehensive review of these techniques see Nichols 2006 [18]). Most of these are publically available from stock centres such as the Bloomington *Drosophila* Stock Centre. One of the most commonly used genetic tools in *Drosophila* is the bipartite UAS/GAL4 tissue-specific expression system. GAL4 is a yeast transcription factor that transactivates the expression of a gene placed downstream of its cognate DNA sequence, the upstream activation sequence (UAS). This allows one to drive the expression of any gene (placed in a transgene downstream of a UAS sequence and a basal promoter) in a cell/tissue-specific manner when GAL4 is expressed in that cell/tissue (under the control of a cell-specific promoter) [19]. An enormous number of cell/tissue-specific GAL4 driver transgenic flies have been created that can be used to express *UAS-transgenes*, including *UAS-tau*, in single cells, cell subsets, or entire tissues. More recently, variations of this expression system have been developed which give the experimenter increased resolution to regulate gene expression in defined cells/tissues (e.g., Split GAL4 and Intersection GAL80/GAL4 systems [20, 21]) and also allow for combined spatial and temporal control of gene expression (e.g., TARGET [22] and GeneSwitch [23, 24]). The TARGET system has been recently employed in *Drosophila* models of tauopathy to study the significance of tau aggregation [25]. In brief, the TARGET system uses a temperature-sensitive variant of the yeast GAL4-repressor, (GAL80^TS^), expressed in all cells by a ubiquitous tubulin promoter. As *Drosophila* does not regulate its body temperature, incubation temperature can be used to control tissue-specific GAL4 activity. At 29-30°C, GAL80^TS^ is deactivated (in restrictive conditions) and permits GAL4-mediated expression of *UAS-transgenes*. At 18°C in contrast, GAL80^TS^ functions normally (in permissive conditions) and potently represses GAL4/UAS activity [22]. Using this system, Colodner and Feany [25] initially reared *UAS-tau* transgenic flies at 29°C to restrict GAL80^TS^ and permit GAL4/UAS-mediated expression of tau in order to allow the formation of tau aggregates. Subsequently, flies were transferred to a GAL80^TS^ permissive temperature of 18°C to block continued GAL4/UAS-mediated tau expression. The authors were thus able to investigate the long-term pathological consequences of tau aggregates by stimulating their formation and assessing neurodegeneration at time points beyond the block on further tau expression. This approach allowed them to draw important conclusions pertinent to aggregate-based disease-modifying therapies [25] (discussed in Section 2). Another very valuable tool in the repertoire of the UAS/GAL4 expression system is the recent generation of *UAS-dsRNAi* responder lines which have been made to more than 90% of the entire fly genome (see Table 1; collections available at the Vienna *Drosophila* Research Centre, The Harvard *Drosophila* RNAi Resource Project, and the NIG-FLY Stock Centre). These flies can be crossed with *UAS-tau*-expressing transgenic flies to enable tissue-specific knockdown of proteins that may interact with tau in order to dissect their role in mediating tau pathogenesis. This system can be used in conjunction with the TARGET system to provide additional temporal control over protein downregulation. The *UAS-dsRNAi* technology can also be used in unbiased studies in which the function of unknown genes that have appeared in human genetic association studies can be rapidly tested. Even before this technology was developed, there was precedence for this approach when mutations in presenilins were associated with familial forms of AD, and the function of presenilin was uncovered in *Drosophila* and *C. elegans* by studying the invertebrate homologue [26]. Functional screening, in *Drosophila* models of tauopathy of loci, identified in human genomewide association studies has already begun [27].

An additional highly valuable resource for tau biologists using *Drosophila* is the vast number of mutant fly stocks which have been generated as a result of the extensive use of *Drosophila* over decades. These stocks can allow tau biologists to ask hypothesis-driven questions about pathways which may be implicated in the pathogenesis of tauopathies. For example, tau is hyperphosphorylated in all tauopathies and one of the kinases believed to be responsible for this aberrant phosphorylation is glycogen synthase kinase beta (GSK-3*β*) (reviewed in [73]). Clearly, an understanding of how pathways regulating GSK-3*β* activity become dysregulated is necessary. In this context, *Drosophila* provides valuable tools because many mutant alleles of fly GSK-3*βshaggy*—*sgg*) have been generated as well as alleles in the *wingless* and insulin signalling pathways within which GSK-3*β* functions. Numerous studies have taken advantage of these mutants by crossing them with transgenics expressing wild-type or mutant human tau and assessing the impact on downstream tau phosphorylation, toxicity, and dysfunction [29, 74]. Not only have such studies highlighted the importance of such pathways in mediating tau phenotypes, but they have also paved the way for exploring disease-modifying therapeutic interventions [47].

If for any reason a novel transgenic fly needs to be generated, this is a relatively straight forward, rapid, and inexpensive task. The transgenesis process (described in more detail in Pandey and Nichols 2011 [12]) usually takes less than 6 weeks and is highly cost-effective. Novel tau transgenic flies have been generated and used. For example, the Feany laboratory and others have generated a number of fly lines expressing human tau with point mutations at various phosphorylation sites, as a tool to investigate the importance of these sites for tau toxicity [30, 31, 75].

Overall, the sophisticated genetic tools together with the vast number of mutant fly stocks that already exist make it relatively easy to study tauopathies in *Drosophila*. Using this system, it is possible to genetically manipulate virtually any physiological pathway-implicated tauopathies and examine their role in the disease process.

#### 1.1.3. *Drosophila* Is Relatively Simple and Yet Participates in Complex Behaviours

Another outcome of the extensive use of *Drosophila* as a laboratory model is that much is known about its anatomy and physiology. It is clear that though this organism is more complex than the other commonly used invertebrate model, *C. elegans*, it is still somewhat simple, both at the cellular and molecular level, when compared to higher vertebrate models. For example, *Drosophila* has fewer isoforms of many proteins (there are 6 isoforms of tau in humans, 3 in rodents, and only 1 in *Drosophila* [76]). The organisation of *Drosophila's* neuronal systems is also relatively simple. For example, the neuromuscular system of *Drosophila* larvae comprises 30–40 motor neurons that innervate 30 muscles per hemisegment, in 12 mostly identical segments [77]. Also, the neuromuscular junctions (NMJs) are easily visualised and well-studied structures (reviewed in Budnik and Gramates 1999 [78]). Such simplicity makes *Drosophila* amenable to experimentation at the level of individual easily identifiable cells whilst still enabling the investigation of functional (including behavioural) outcomes of such manipulations. We have previously utilised this simple NMJ system of *Drosophila* larvae to investigate the effects of highly phosphorylated tau on the structure and function of the NMJ on muscle 4 [48].

Despite its relative simplicity compared to other model organisms, *Drosophila* is capable of participating in “higher complex behaviours” such as courtship behaviour [79], learning and memory [80], social interactions [81], aggression [82], grooming [83], and even alcohol preference and addiction [16]. This makes it amenable to cognition-centred functional assays that are particularly relevant when modelling human neurodegenerative diseases like Alzheimer's disease, characterised by progressive loss of complex cognitive processes such as learning and memory. Indeed, some groups have utilised learning and memory assays to assess the impact of tau expression in their *Drosophila* models of tauopathy; Mershin et al., demonstrated that associative learning and memory processes were significantly compromised in transgenic flies expressing human tau, bovine tau, or even extra copies of *Drosophila* tau [51]. These results clearly showcase how this experimental paradigm can be used for dissecting the mechanisms responsible for learning and memory defects in the early stages of tauopathies like Alzheimer's disease.

#### 1.1.4. Imaging Physiological and Pathological Events *In Vivo* in *Drosophila*

*Drosophila* larvae have a clear cuticle and the musculature beneath is richly innervated with a well-characterised network of motor and sensory neurons. It is therefore possible to visualise, in real time, the activity of any physiological or pathological process that occurs within these neurons as long as one is able to fluorescently tag one of the proteins that is participating in that cellular process. We exploited this attribute of *Drosophila* when we tested the long-standing “tau and microtubule” hypothesis in our *Drosophila* model of tauopathy. This hypothesis stemmed from observations made in *in vitro* experimental paradigms which led to the suggestion that hyperphosphorylated tau exhibits reduced microtubule binding and would result in breakdown of cytoskeletal integrity and disruption of axonal transport (reviewed in Cowan et al., 2010 [84]). The rodent models of tauopathy that existed at that time were not amenable to analysis of axonal transport *in vivo* so our study in *Drosophila* was the first to test this hypothesis *in vivo*. We visualised axonal transport in real time in living intact third instar larvae, by using the UAS/GAL4 system to drive expression of GFP tagged neuropeptide Y and a wild-type isoform of human tau (0N3R- which is constitutively highly phosphorylated) within the motor neurons that run beneath the transparent larval cuticle. As predicted by the “tau-microtubule hypothesis”, we found that the expression of highly phosphorylated tau led to a breakdown of microtubular integrity, causing axonal transport disruption, synaptic dysfunction, and locomotor impairment [47, 54]. Thus by using *Drosophila*, we were able to test all aspects of this hypothesis *in vivo* for the first time.

#### 1.1.5. *Drosophila* Is Amenable to Medium-to-High Throughput Enhancer/Suppressor Screens

It is relatively simple to establish an enormous number of different crosses between flies. This, coupled with the fact that there are numerous readouts of tau-mediated neuronal toxicity and dysfunction in *Drosophila* models of tauopathy, makes *Drosophila* particularly well suited to unbiased, *in vivo* genetic, or pharmacological enhancer/suppressor screens. The advent of digital tracking technologies which enable one to track behavioural parameters of *Drosophila* adults and larvae in a *semiautomated* manner [85] should make *Drosophila* models even more amenable to medium-to-high throughput screening. As discussed in Section 1.2.2, the genetic enhancer/suppressor screens that have been conducted on *Drosophila* models of tauopathy have identified both previously suspected proteins (like PAR-1 kinase—[32]) as well as novel modifiers (such as components of the JAK/STAT family [25]). Extensive pharmacological enhancer/suppressor screens have not been reported in *Drosophila* models of tauopathy, but have been carried out to identify a handful of lead compounds in *Drosophila* models of Huntington's Disease and other Fragile X syndrome (reviewed in Newman et al., 2011 [86] and Pandey and Nichols 2011 [12]). We have recently carried out a targeted pharmacological enhancer/suppressor screen to validate the modulatory role of a few compounds believed to interfere with protein-folding pathways implicated in tauopathies [85]. 

### 1.2. *Drosophila* Models of Tauopathies (Summarised in Table 1)

#### 1.2.1. How Have Tauopathies Been Modelled in *Drosophila*?

Tauopathies are characterised by aggregates of abnormally phosphorylated and misfolded wild-type (wt) or mutant tau. To model tauopathies therefore, most studies have utilised the UAS-GAL4 expression system to target the expression of either wt or mutant human (and even bovine, rodent, and *Drosophila*) tau to specific neuronal or glial cells in both larvae and adult *Drosophila*. In many of these studies, the human tau expressed is highly phosphorylated, with some studies reporting that it also becomes misfolded and/or insoluble [33, 34, 53]. In general, the tau does not seem to aggregate into bona fida, EM-verified tau filaments, unless it is coexpressed with an enhancer [29], or expressed in glia [25], (see Section 2.2). The consequences of tau expression have then been investigated both by assessing neuronal function (including axonal transport, synaptic function, olfactory learning, and locomotor behaviour) and neuronal death (in assays such as the rough-eye phenotype, CNS neurodegeneration, and loss of notal bristles).

#### 1.2.2. How Have *Drosophila* Models Been Used to Dissect Disease Mechanisms? 

Some of these models have been used in unbiased genetic enhancer/suppressor screens to identify proteins that interact with tau. Shulman and Feany were the first to report the results of a forward genetic screen looking for modifiers of the rough eye phenotype induced by the expression of V337M human tau [32]. In this screen, they identified both enzymes already implicated in tau phosphorylation (like PAR-1 kinase and PP2A phosphatase) and apoptosis as well as proteins that had never previously been implicated in tauopathies. These included *Drosophila *homologs of mammalian proteins such as Ataxin-2 and Glypican as well as cytoskeletal proteins Filamin and MAP1b. Four synaptic cytoskeletal components were also identified in a similar enhancer/suppressor screen together with molecular cochaperones, a tyrosine phosphatase, ion transporting ATPases, and RNA binding proteins as well as transcriptional cofactors [35]. Again, though some of these were expected to modulate the tau phenotype, others were novel modifiers, whose role in the disease process has still not been thoroughly examined.

These enhancer/suppressor studies are often accompanied by complimentary experiments in which the identified genes are either coexpressed with human tau [32, 34], or their endogenous orthologs are genetically or pharmacologically suppressed. This enables the verification of their role in tau-mediated toxicity. For example, in their screen, Colodner and Feany identified a suppressor of JAK/STAT signalling as a modulator of glial tau toxicity in their model. They went on to verify this by demonstrating that coexpression of activators of JAK/STAT suppressed, whereas inhibitors of JAK/STAT enhanced glial-tau-mediated toxicity [25].

In some studies, the *Drosophila* tauopathy models were used in biased hypothesis-led experiments to dissect mechanisms by which tau mediates dysfunction or toxicity. These included testing of hypotheses about abnormal tau-driven loss of function or gain of toxic function and the role of tau phosphorylation and aggregation in these pathogenic events. These studies investigated the effects of abnormal tau on (a) microtubular cytoskeletal structure and function [36, 37, 54], (b) on synaptic structure and function [34, 48], (c) neurite outgrowth [38], (d) endogenous proteins including *Drosophila* tau [54], (e) lysosomal function [39, 55], and (f) the cell cycle [40]. They have also highlighted the relationship between abnormal tau and oxidative stress [56] and alluded to mechanisms of tau turnover [34, 57, 87]. Some studies have also investigated the relationship between tau and A*β* in *Drosophila* [41, 58, 74, 88]. These studies have provided valuable insights into the possible mechanisms by which abnormalities in tau cause neurodegeneration in tauopathies.

## 2. What Insights About Tauopathy Have We Gained from *Drosophila* Models?

One of the critical obstacles to developing disease-modifying therapies for the treatment of tauopathies is our incomplete understanding about disease pathogenesis. *Drosophila* models of tauopathy have provided insights into some of these pathogenic processes including mechanisms of toxicity, mechanisms of tau turnover, and identification of pathways by which tau and A*β* may interact. They have also raised questions about the significance of tau aggregation. Generally, these insights add to those obtained from similar rodent models of tauopathies but in some instances *Drosophila* models, by virtue of the elegant genetics tools available, are able to more tightly correlate pathogenic events to causative changes in tau. The fact that *Drosophila* models are amenable to genetic enhancer/suppressor screens, has further enabled the identification of novel players in the disease processes.

### 2.1. Mechanisms of Tau Toxicity

#### 2.1.1. Expression of Abnormal Tau Causes Neuronal Death

The expression of wild-type or FTDP-17 mutant human tau in the *Drosophila* eye causes cell death which gives rise to the well-characterised “rough-eye phenotype” [32, and others]. Neuronal death following the expression of wild-type (0N4R and 2N4R) or mutant (V337M and R406W) human tau in other neuronal populations including cholinergic neurons, sensory neurons (including notum bristles), and mushroom bodies has also been reported [38, 50, 53, 59]. In some studies, human tau expression has been limited to specific neuronal or glial populations, whereas in others it is expressed panneuronally or expressed in both neurons and glia. Irrespective of the cellular population the tau is expressed in, neurotoxicity has been reported in many of the models in both a cell autonomous and nonautonomous manner. This toxicity is unlikely to be simply because the tau is human, as similar neurodegeneration has been reported following the expression of bovine tau, rodent tau, or even overexpression of *Drosophila* tau [42, 50, 59]. This suggests that excessive or misexpression of tau proteins is toxic, irrespective of the host species from which the tau is derived. One might speculate as to why expression of wild-type (as opposed to disease causing mutant) tau should cause toxicity—tau is normally expressed in all neurons and its presence is not toxic in normal healthy cells. Though there is clearly a dosage effect [49], an imbalance in the normal tau isoform ratios following the expression of exogenous tau may be one cause of toxicity because some FTDP-17 splice mutations cause neurodegeneration simply by altering the ratio of 3R to 4R tau isoforms in the brain. It is also possible that the phosphorylation state of tau changes when its expression equilibrium is changed and since this has been shown to be critically involved in the mechanisms by which tau causes toxicity (and dysfunction), as discussed below, this may provide one explanation for why ectopic expression of wild-type tau leads to neurodegeneration. In this regard, these models recapitulate sporadic tauopathies where the trigger for the conversion of normal wild-type tau to a hyperphosphorylated and misfolded state is not clear.

The cellular mechanism by which high tau expression causes neuronal death is not entirely understood. However, more than one toxic mechanism may play a role since a variety of morphological changes have been described in the dying human-tau-expressing cells. Affected neurons have been shown to exhibit signs of both necrotic and apoptotic degeneration. Williams et al., (2000) expressed various tau transgenes (human wild-type 0N3R, bovine, and rodent) in larval sensory neurons and reported degeneration characterised by abnormal axon bundling, reduced arborisation, axonal swelling, and beading; in severely affected animals there was also a clear loss of axonal projections [50]. A number of studies expressing wild-type or mutant 4R tau have reported vacuolization and abnormal swelling of nuclei in adult brain and retina [29, 40, 51, 53]. There is also evidence that apoptosis occurs in the human-tau-expressing cells. TUNEL-positive cells have been reported in models expressing wild-type and mutant tau in neurons and glia [25, 40, 43]. Furthermore, coexpression of two inhibitors of apoptosis, p35 and *thread*-suppressed V337M-mediated rough-eye phenotype, whilst the *Drosophila* homology of Fem1, an apoptosis activator exacerbated this phenotype in two different *Drosophila *models of tauopathy [32, 42]. To identify how aberrant tau triggers apoptosis, Khurana et al., (2006) demonstrated that cell cycle activation occurs in the brains and eyes of transgenic mice expressing wild-type or mutant human tau, and that this occurs downstream of tau phosphorylation and activates apoptosis [40]. Collectively, all these studies indicate that activation of apoptosis may be one of the mechanisms by which tau causes neurodegeneration. Apoptotic cell death is also implicated in the pathogenic mechanisms of other chronic neurodegenerative diseases like Parkinson's disease and Huntington's disease.

Like apoptosis, oxidative stress is believed to play a role in many chronic neurodegenerative diseases. Studies in *Drosophila* imply that it may also be involved in the mechanism by which aberrant tau mediates toxicity in tauopathies. Dias-Santagata et al., (2007) [56] demonstrated that partial inactivation of antioxidant pathways in flies expressing R406W tau exacerbated the neurodegenerative phenotype in their model. Moreover, the antioxidant Vitamin E suppressed, whilst treatment with the prooxidant mitochondrial toxin paraquat, exacerbated these phenotypes. These results suggest that the expression of aberrant tau triggers oxidative stress which may then contribute to the mechanism by which the tau expressing neurons are degenerating.

It has traditionally been thought that the aggregates of tau (filaments and tangles) are in themselves toxic and thus are responsible for neurodegeneration in tauopathies. This view is now being challenged (Section 2.2). However, since glial expression of human tau in one *Drosophila* model of tauopathy led to the formation of glial tangles, it is conceivable that these were responsible for the tau-mediated neurodegeneration described in that model [25]. However transferring these flies to a temperature which repressed tau expression, led to a dramatic reduction in the number of TUNEL-positive cells without any change in the numbers of glial tau tangles. This suggests that the aggregated tau is not responsible for the tau toxicity observed in this model. A similar observation has been made in a rodent model of tauopathy—discussed in greater detail in Section 2.2 [89]. Even if aggregated tau does not play a critical role in tau-mediated toxicity in *Drosophila*, misfolded (but soluble) tau may be involved. Bilen and Bonini (2007) demonstrated that components of the chaperone and ubiquitin proteosome pathways [44], which are known to interact with misfolded tau [90], potently suppressed the rough-eye phenotype induced by either wild-type human tau or R406W tau. This implies that the misfolded tau was mediating the toxicity in their model. Further support for this idea comes from observations made by a few groups that when the expression of human wild-type or mutant tau causes the rough-eye phenotype, it is often MC1 or Alz50 positive—both of these antibodies react with misfolded species of tau [53]. Thus it would seem that, at least in *Drosophila*, tau does not need to aggregate to cause degeneration. This raises the intriguing possibility that tau aggregation is a late-stage event that begins after the onset of neurodegeneration in tauopathies.

Another potential mechanism responsible for tau toxicity is the displacement of tau from the axon to the soma. Tau is classified as an axonal protein and its displacement into the somatodendritic compartment is often considered by some to be a pathogenic event culminating in tangle formation and degeneration. One study from a *Drosophila *model of a familial form of Parkinson's Disease (PD) (which is a secondary tauopathy since tau aggregates are often found in this condition) lends support to this idea. In this model, the expression of a disease causing mutant kinase (leucine rich repeat kinase 2 or LRRK2) in adult brain dopaminergic neurons causes degeneration of dendritic arborizations, which is associated with a mislocalisation of the endogenous *Drosophila* tau into the soma [52]. Furthermore, suppression of endogenous *Drosophila* tau using RNAi suppresses, whereas exogenous overexpression of human wild-type tau exacerbates, the mutant LRRK2-mediated dendritic degeneration. Similar results were reported by Zempel et al., in primary culture [91]. They showed that degeneration of dendritic spines following the exposure to A*β* oligomers was associated with mislocalisation of tau from the axon to the soma. Collectively, these results suggest that mislocalisation of tau can cause degeneration. This may be one mechanism responsible for degeneration in the *Drosophila *models of tauopathy described above.

The role played by tau phosphorylation in mediating tau toxicity has also been investigated in most of the *Drosophila *models of tauopathy. In one of the early reports of tau-mediated degeneration in the *Drosophila* nervous system, Wittmann et al., reported a concomitant increase in tau phosphorylation at the AT8 and AT100 sites [53]. This implied that phosphorylation of tau occurs during tau-mediated degeneration. Jackson et al., then demonstrated that the loss of function of the *Drosophila* homolog of the tau kinase GSK-3*β*, (*shaggy) sgg*, exacerbated, whilst the coexpression of *sgg* with tau enhanced the human tau rough eye phenotype [29]. Similarly, Yeh et al., [38] showed that the loss of notal bristles in their model was diminished when human tau was expressed together with a dominant negative *sgg*. This implicates tau phosphorylation causally in the degenerative process. This idea was corroborated by the unbiased findings of the genetic enhancer/suppressor screen carried out by Shulman and Feany in 2003, in which it was seen that the largest class of modifiers of the V337M-induced rough eye phenotype were tau kinases and phosphatases [32]. However, the conclusive proof for the critical role played by tau phosphorylation in tau-mediated neurotoxicity was provided by Nishimura et al., in 2004 who employed fly genetic tools to tightly correlate tau phosphorylation with degenerating neurons [43]. Like Jackson et al., they first demonstrated that the coexpression of a tau kinase (in this case PAR-1 kinase, the *Drosophila *homolog of another tau kinase MARK), with either wild-type or R406W mutant human tau increases its phosphorylation at Ser^262^/Ser^356^ (the 12E8 site) and exacerbates the tau-mediated rough-eye phenotype. They then went on to confirm that PAR-1 phosphorylation of tau is essential for its toxicity by demonstrating that tau nonphosphorylatable at this site (4R tau with alanine substitutions at Ser^262^/Ser^356^) does not cause degeneration in the eye [43] (this result was recently reproduced by Chatterjee et al., [37]). Finally, they used the MARCM expression system to generate PAR-1 mutant clones in the human-tau-expressing flies and showed that most of the cells that were TUNEL positive were in the PAR-1/human tau clones (which were also 12E8 positive) [43]. Fulga et al., added further weight to the role of tau phosphorylation in tau toxicity by showing that the expression of a pseudophosphorylated tau mutant (4R^E14^) was more toxic, whilst a nonphosphorylatable mutant (4R^AP^) was less toxic to retinal photoreceptors [30]. Tau phosphorylation is also implicated in tau-mediated degeneration of other CNS tissues. In the model used by Colodner and Feany (2010), in which human wild-type tau is expressed in glia, there is an age-dependent increase in tau phosphorylation at some (AT8 and AT100) epitopes which coincides with the increased tau insolubility/glial tangle formation and apoptotic cell death they observe [25]. Kosmidis et al., showed that the 0N4R tau-mediated ablation of mushroom bodies in the adult brain (due to a toxic effect of tau on mushroom body precursors) was enhanced by the expression of a phospho-mimicking tau mutant (0N4R^E14^) and suppressed by expression of a nonphosphorylatable tau mutant (0N4R^AP^) [59]. Aberrant tau phosphorylation is also believed to play a role in the mislocalisation of tau from the axonal to somatodendritic compartments [52, 91]. Lin et al., showed that the degeneration of dendrites which occurred when tau mislocalised into the somatodendritic compartment in their *Drosophila *model, was associated with increased AT100 (Thr^212^, Ser^ 214^) immunoreactivity. Moreover, the expression of a phospho-mutant form of human tau which is not phosphorylatable at the AT100 site led to an abrogation of the dendritic degeneration phenotype and thus causally linked tau phosphorylation at this site with the dendritic toxicity they observed [52].

In addition to the mechanisms discussed above, other novel pathways and cellular processes may be involved in mediating tau toxicity. Some of these have been identified from genetic enhancer/suppressor screens and include the JAK/STAT pathway [25]. This pathway is involved in a variety of cellular processes including the transduction of inflammatory signals. At the moment, the mechanism by which this pathway mediates tau toxicity is unclear. Nonetheless, it would seem that glial tau expression leads to suppression of this pathway and that tau toxicity can be rescued by increasing the expression or activity of components of this pathway. Further investigation into this pathway and the role it plays in tau-mediated toxicity may lead to novel, glial-based therapeutic interventions not just for glial cell centred tauopathies, but also for other tauopathies. A second group of proteins that have previously not been extensively studied in the context of tau-mediated toxicity are components of the actin cytoskeleton. These were identified in the enhancer/suppressor screen carried out by Blard et al., in 2006 [34]. Subsequently, Fulga et al., demonstrated that human R406W tau interacts with F-actin, leading to its abnormal bundling and accumulation, and that genetic reduction of actin suppressed, whilst the overexpression of actin exacerbated the V337M or wild-type 4R-mediated rough-eye phenotype in their *Drosophila* model [30]. Thus, crosstalk clearly occurs between constituents of the axonal microtubular and synaptic actin cytoskeleton, but how this is affected when these components become abnormal in tauopathies is unclear. This is certainly an area worthy of investigation since it may be critically involved in the mechanisms giving rise to the synaptic dysfunction/loss which is evident in the early stages of tauopathies [92]. Indeed components of the actin network have been shown to colocalise with NFTs in both AD and FTDP-17 brains [93].

#### 2.1.2. Expression of Abnormal Tau Causes Neuronal Dysfunction

Though, as discussed above, there are many *Drosophila *models demonstrating that expression of abnormal tau is associated with neurotoxicity, there are other *Drosophila *models which report behavioural phenotypes in the absence of overt neurodegeneration. We have shown that expression of wild-type human (0N3R) or *Drosophila* tau in larval motor neurons causes neuronal dysfunction. This is characterised by disruption of axonal transport and synaptic structure and function [47–49, 54]. In our model, there was no evidence of tau aggregation or neurodegeneration so the tau-mediated neuronal dysfunction was caused by a soluble tau species. Like us, Falzone et al., also report axonal transport defects following expression of human mutant (R406W) tau in their *Drosophila *model, which was seen in the absence of neuronal death or tau aggregation [60]. Analogous results have emerged from the laboratory of Skoulakis who report significant learning and memory deficits in transgenic flies expressing wild-type human tau (2N4R), bovine tau, and *Drosophila* tau, before any evidence of neurodegeneration [51] (Mershin et al., 2004). Similar findings have been reported in a rodent model of tauopathy in which aged mice expressing wild-type human tau (2N4R) exhibited learning and memory impairments but in the absence of overt neuronal loss [94].

Almost all the studies in which a soluble tau species is associated with neuronal dysfunction in the absence of degeneration implicate the phosphorylation state of the tau in the causative mechanism. In our studies, we found that reducing tau phosphorylation (at the PHF-1 and AT8 sites) by treatment with LiCl suppressed, whilst increasing tau phosphorylation by the coexpression of *sgg,* enhanced the axonal transport and locomotor defects [47]. Kosmidis et al., (2010) showed that the expression of a human 4R tau phospho-mutant (2N4R^STA^ which cannot be phosphorylated at Ser^238^ and Thr^245^) which is highly phosphorylated at the AT100, AT8, pS262, and pS356, leads to profound memory impairments in the absence of mushroom body degeneration [59]. The results from rodent models of tauopathy (such as Kimura et al., 2007 [94] described above), also suggest that hyperphosphorylated tau plays a causal role in mediating memory impairments seen in the absence of cell death or tangle formation.

The mechanism by which highly phosphorylated tau disrupts neuronal function is likely to involve a phosphorylation-mediated reduced ability of hyperphosphorylated tau to bind to and stabilise microtubules. Highly phosphorylated tau has been shown to have a reduced ability to bind to microtubules *in vitro* [95]. In agreement with this, we found that the breakdown of cytoskeletal integrity evident in our *Drosophila* model occurred because the highly phosphorylated (AT8 and PHF-1 positive) human (0N3R) tau that we expressed was compromised in its microtubule-binding ability [54]. Others too have shown that a significant proportion of the human tau that is expressed in their *Drosophila *model of tauopathy is not bound to microtubules because it is highly phosphorylated [36]. Since highly phosphorylated (AT8 positive) tau is evident in pretangle neurons in AD brains, it is conceivable that the microtubular cytoskeleton and thus axonal transport in these neurons are compromised [96, 97]. This may be responsible for the neural network disruptions that manifest as learning and memory impairments in the early stages of disease.

An unexpected additional pathogenic effect of soluble highly phosphorylated human tau that we uncovered in our model is that it binds to the endogenous *Drosophila* tau and compromises its microtubule-binding ability as well. Thus, the breakdown of cytoskeletal integrity that we reported possibly occurred because of functionally incompetent highly phosphorylated human tau *and* the pathogenic conversion of normal endogenous tau [54]. This ability of abnormally phosphorylated soluble tau to bind to and functionally compromise other proteins has been demonstrated in a cell culture experimental model [98].

### 2.2. Significance of Tau Aggregation in Relation to Tau Toxicity

Although misfolded protein aggregates characterise many common proteinopathies including Alzheimer's Disease, Parkinson's Disease, and Huntington's Disease, the role that they play in the disease process is debatable. Emerging evidence from various models of these diseases suggests that there is a dissociation between the aggregates themselves and the underlying toxicity, and that instead the precursors of the aggregates may be the toxic species. *Drosophila* models of tauopathies have certainly contributed to this argument by demonstrating that degeneration occurs in the absence of overt tau aggregation. Williams et al., reported degeneration of sensory neurons following expression of human, bovine, or rodent tau in the absence of tangle formation [50]. Subsequently, Wittmann et al., reported neurodegeneration following expression of both human wild type and FTDP-17 mutant tau, but here too in the absence of tau aggregates [53]. These findings imply that in these models of tauopathy, dysfunction and toxicity may be caused by a soluble hyperphosphorylated tau. Virtually, all *Drosophila *models of tauopathy have provided further proof for this concept because in the majority of studies which have expressed some form of tau in *Drosophila*, no insoluble tau has been detected. We would argue that this does not necessarily indicate that *Drosophila* provides a poor model of tauopathy; rather these studies furnished evidence that tau aggregation is not necessary for tau toxicity ([50, 53] and extended by many others). This conclusion has been backed up by rodent studies. For example, mice expressing wild-type human tau show behavioural deficits without NFTs [94], and the triple-transgenic mice (expressing tau, APP, and presenilin-1) also display neuronal dysfunction before the formation of NFTs (and indeed before amyloid plaques and cell death) [99]. Perhaps ironically, *Drosophila* models have also provided insights into the mechanism by which tau causes dysfunction early in the disease process, without the “confounding” factors of insoluble tau and cell death that are often encountered in rodent models. In addition, as has been discussed elsewhere in this paper (see Section 1.1), expressing different forms of tau in specific neuronal populations using the UAS/GAL4 system provides numerous models to answer very specific questions, without the need (or ability) for any one of these models to fully recapitulate all aspects of disease.

One might wonder why there is no insoluble tau in most fly models. In the case of models expressing tau in motor neurons with the D42 driver [47–49, 54], in sensory neurons [50], or in the mushroom body region of the brain [51] the lack of insoluble tau might possibly be because these models represent early neuronal dysfunction: if flies lived longer, perhaps tangles and cell death would eventually occur. However, in the case of models expressing tau in photoreceptor neurons with the GMR, Sevenless or EY driver [29, 32, 36, 39, 40, 43, 100] or in cholinergic neurons (Cha driver) [53], or panneuronally with Elav driver [40, 53, 56] this is clearly not so, because neurons die within the fly's lifetime without ever forming NFTs. In these retinal cell death models, it could be suggested that perhaps flies lack the molecular mechanisms necessary to create tangles. Again, we know this is not so because the co-overexpression of the tau kinase *shaggy* can force the production of tangles, even in this retinal system [29], and because tau tangles can be formed in glial cells of the fly [25].

As is implicit in the preceding paragraph, one key phenomenon of tau toxicity that has been evident in fly studies is the cell-type specificity of tau's effects on neurons. It has long been apparent from the human conditions that some cell types are more susceptible to tau pathology than others [101]. Furthermore, human tauopathies and numerous *in vitro* studies have shown that tau of different isoforms and different phosphorylation states (not to mention different solubility states) can all behave quite differently [102]. These phenomena can be investigated precisely using the UAS/GAL4 system in *Drosophila*, as the Skoulakis laboratory is doing, in a directed manner that is rather difficult in rodents. For example, in one study, they directly compared the effect of taus from different species expressed in mushroom body neurons on functional learning and memory tasks—discussed in Section 2.5 [51]. They have expressed a variety of disease-mutant tau constructs in a variety of neuronal cell types [45], and discovered that the taus are processed and posttranslationally modified quite differently in different neuronal types, as a function of the regulation of kinases there. They subsequently found that mushroom body neurons are more susceptible to wild type tau than other neurons, but less susceptible to some of the disease mutant taus [59]. Since tau aggregation can be induced genetically in flies by coexpression of the relevant kinases, one could use these genetic tools in *Drosophila *to similarly investigate isoform and cell-type differences in susceptibility to tau aggregation.

Where insoluble tau has been detected in fly, it does not appear to be detrimental. In Chau et al.'s study older tau-expressing flies developed insoluble tau (the precise nature of which was unclear from their report) at 22 days of age; their rough-eye phenotype was no worse than young flies without insoluble tau [33]. This might suggest that the aggregates per se are not detrimental. Dramatic and convincing evidence that aggregated tau is not the major toxic species has come from mice, in which reducing tau expression conditionally in symptomatic mice can ameliorate the phenotype even though tau tangles remain behind [89]. A similar approach was recently taken in *Drosophila*, in a model in which wild-type tau expression in glia causes neurofibrillary tangles in glia and cell death of glia and neurons [25]. In this study, when tau expression was turned off subsequent to tangle formation, cell death was prevented though the tangles remained.* Drosophila* might be a useful system to extend these findings because of the possibility (through the TARGET system and the of attB/attP integration system) of replicating this experiment while also directly comparing many species, isoforms, or alleles of tau.

Taken together, these results from the *Drosophila* models of tauopathy are in agreement with the results obtained from rodent models and cast doubt about the requirement of tau aggregation in the pathogenesis of tauopathies. Instead, they imply that a highly phosphorylated, soluble species of tau is critically involved in the mechanisms that lead to neuronal dysfunction and degeneration. Whether this represents the state of affairs in the early stages of disease only, and a second set of degenerative events stimulated by tau aggregation takes effect at later stages of disease is yet to be determined.

### 2.3. Turnover of Tau

Protein quality control features prominently in the discussion surrounding tauopathies and other neurodegenerative diseases collectively termed “foldopathies”. Protein quality control can be broadly thought to encompass both systems that are activated when proteins misfold, (such as the chaperone family and the unfolded protein response (UPR)), as well as those that deal with the clearance of terminally misfolded or aggregated proteins (such as the autophagic/lysosomal systems and the ubiquitin proteosome pathway (UPS)). *Drosophila* models of tauopathy have demonstrated that tau interacts with all of these pathways and that they may play a pivotal role in its turnover in both normal and disease situations. These discoveries not only impart information about the role of protein quality control pathways in proteinopathies, they also highlight potential therapeutic targets.

#### 2.3.1. Tau May be Degraded By the Autophagy/Lysosomal Pathway

It was suggested that tau could be cleared by the autophagic/lysosomal pathway when Berger et al., demonstrated that Rapamycin, a known inducer of autophagy, marginally suppressed the tau-mediated rough eye phenotype [100]. However, since the authors could not detect human tau in their flies (possibly because of a weak driver), they could not directly relate the suppression of tau phenotype with autophagic clearance of tau. The more conclusive proof that tau may be turned over by this pathway and that a dysfunction in this process could play a role in tau-mediated toxicity came from the results of Dermaut et al., [39] and Khurana et al., [55]. Both groups demonstrated that lysosomal dysfunction, either through a loss of function mutation of an endogenous lysosomal protein [39], or a genetic reduction of the lysosomal enzyme cathepsin D, potently exacerbated the human-tau-mediated phenotypes in their respective models. Khurana et al., then went on to show that though this lysosomal dysfunction was not associated with an increase in the total tau levels, nor a change in the phosphorylation state of tau, there was a five-fold increase in the amount of caspase cleaved N terminal truncated tau [55]. This form of tau was much more toxic and insoluble than full length wild type or mutant tau. These results suggest the tantalising possibility that normal lysosomal function prevents tau from being cleaved by caspases and thus prevent it from acquiring a more pathogenic, aggregation-prone status. Whether lysosomal function in healthy neurons protects tau by turning it over in a normal pathway with little tau getting shunted down a “caspase cleavage pathway” is not clear. Since both dysfunctional lysosomes and truncated tau species have been detected in AD brains (reviewed in Khurana and Feany (2007) [103]), these findings provide an insight into potential mechanisms that may trigger tau aggregation in tauopathies.

#### 2.3.2. Tau Abnormalities May Trigger the Unfolded Protein Response

The unfolded protein response (UPR) deals with excess misfolded protein in the ER and aims to restore homeostasis by reducing entry of such proteins into the ER, stimulating the degradation of the misfolded/aggregated proteins, or upregulating heat shock proteins (HSPs) and other protein quality control genes. Loewen and Feany (2010) recently demonstrated that the UPR is activated in transgenic flies expressing human wild-type and mutant tau, and that there is a positive correlation between the extent of the UPR activation and the degree of toxicity [57]. The fact that the greatest activation of the UPR was seen in flies expressing a phospho-mimicking mutant form of tau led the authors to speculate that excessively phosphorylated tau was triggering the UPR activation. When components of the UPR machinery were genetically suppressed to prevent its induction, there was a strong exacerbation of the tau-mediated phenotypes. These results imply that the unfolded protein response occurs as a compensatory measure to protect cells against abnormal, hyperphosphorylated, and possible misfolded tau species. There is some evidence to suggest that the UPR is activated in AD [104], but the fact that misfolded and hyperphosphorylated tau species still persist and cause neurodegeneration implies that it cannot adequately deal with the abnormal tau.

#### 2.3.3. The Chaperone/UPS System May Be Involved in the Degradation of Phosphorylated Tau

Though there is a lot of evidence from rodent and cell culture models of tauopathy showing that chaperone proteins such as CHIP (C-terminus of Hsc-70 interacting protein) regulate the turnover of phosphorylated tau via the UPS [105], the evidence from *Drosophila *is conflicting. Feuillette et al., reported that neither genetic downregulation, nor pharmacological inhibition of the UPS increased the total level or phosphorylation state of endogenous *Drosophila* tau. This prompted them to conclude that tau is not degraded by the UPS in *Drosophila *[87]. This was contrary to the findings of Blard et al., a year later, that the inhibition of proteasomal activity in flies expressing wild-type (0N4R) tau resulted in the accumulation of high-molecular-weight tau species [34]. Moreover, in agreement with the findings from rodent and cell culture models, they found that preventing tau phosphorylation by expressing it in a background of dominant negative *sgg* also led to an accumulation of high molecular weight tau species. This implies that UPS-led clearance of tau in their model was dependent upon its phosphorylation state. With regard to the role played by chaperones in the refolding and clearance of tau, the results from *Drosophila* models are perplexing. Shulman and Feany did not identify any member of the chaperone family in their V337M tau-mediated rough eye phenotype enhancer/suppressor screen in 2003 [32]. Nor did they find any modulatory effect of the coexpression of hsp70 and hsc4, two molecular chaperones, on this tau phenotype. In contrast, Blard et al., did identify molecular chaperones in the enhancer/suppressor screen that they carried out three years later. However when these chaperones were coexpressed with V337M, they exacerbated the rough eye phenotype in their model [34]. The significance of these findings is not clear.

### 2.4. A*β* and Tau Interactions

The relationship between the tau and amyloid pathologies and the pathways that facilitate an interaction between them has been a matter of intense debate and scientific research in this field. The general consensus is that amyloid pathology lies upstream of tau pathology and thus must activate cellular processes that cause the hyperphosphorylation and aggregation of tau. The results that have come forth from the few *Drosophila* models of tauopathy that have attempted to study the interaction between these two proteins broadly support this view.

Torroja et al., demonstrated in 1999 that the pan-neuronal expression of either bovine tau or the *Drosophila* homolog of amyloid precursor protein (APPL) results in the retention of vesicles in larval motoneurons [58] (an effect also seen after expression of human APP [106]). This phenotype becomes significantly more severe when both are coexpressed implying that the two proteins interact to cause a disruption of axonal transport. We reported a similar result in 2010, when we found that coexpression of human A*β*_42_ with wild-type human tau (4R) potently exacerbates tau-mediated axonal transport disruption in larval motor neurons [74]. We went on to show that the two proteins interact to impair the locomotor ability of adult flies and shorten their lifespan, and that these exacerbatory effects can be prevented with LiCl. This implies that GSK-3*β* is mediating the interaction between tau and A*β*_42_ in our flies. More recently, Sofola et al., (2010) have provided more evidence to suggest that A*β* may be activating GSK-3*β* in their *Drosophila* model [88]. They used an inducible gene expression system to express Arctic mutant A*β*_42_ specifically in adult neurons and found that it accumulates with age and causes increased mortality and a progressive neuronal dysfunction in the absence of overt cell loss. This also leads to an increase in GSK-3*β* activity. Interestingly, the pathogenic effects of A*β*_42_ in this model are significantly suppressed when A*β*_42_ is expressed in a *Drosophila* tau null line, demonstrating an interaction between the two proteins in the pathogenic process [88]. This result supports the observations made in various rodent models of tauopathy that A*β* requires tau to mediate its pathogenic effects [107] (for a more comprehensive review see Morris et al., 2011 [7]).

Other reports suggest that tau and A*β*/APP may interact via other kinases. Wang et al., (2007) the demonstrated that coexpression of human R406W tau with human APP leads to an exacerbation of the tau-mediated rough eye phenotype which is suppressed by knocking down the tumour suppressor protein LKB1 [46]. The authors suggest that APP activates LKB1 to enhance PAR1-mediated phosphorylation of human tau. This is because the coexpression of tau and APP leads to an increased tau phosphorylation at the PAR1 site 12E8, which is diminished in flies in which LKB1 is knocked down by RNAi. Iijima Ando and Iijima (2010) show that A*β*_42_ exacerbates the rough eye phenotype induced by the expression of human wild type (0N4R) tau, and that this effect is accompanied by an increased phosphorylation of tau at a number of sites including Ser^202^, Thr^231^, and Ser^262^[41]. The authors go on to show that the exacerbatory effect of A*β*_42_ on the tau rough eye phenotype is suppressed if A*β*_42_ is coexpressed with a mutant form of tau which cannot be phosphorylated at Ser^262^. This indicates that A*β*_42_-mediated phosphorylation at this site is critical in its pathogenic interaction with tau. The authors suggest that the damage-activated check point kinase 2, whose RNA levels are upregulated in their A*β*_42_ expressing flies, may be responsible for mediating A*β*_42_'s effects on tau [41].

Overall, the *Drosophila* models of tauopathy in which both tau and A*β*/APP are coexpressed can provide valuable insights into the cellular pathways that mediate the interaction between these two proteins. They can also serve as useful platforms to screen the therapeutic agents designed to interfere with this interaction.

### 2.5. Isoform Specific Differences

Alternative splicing of one gene leads to the translation of 6 isoforms of tau in the adult human brain, which differ in the presence or absence of 1 or 2 N-terminal domains and have either three (3R) or four (4R) C-terminal microtubule binding domains. In the adult brain, the ratio of 3R : 4R isoforms is 1. Rodents have 3 isoforms of tau and *Drosophila* has one. All tau isoforms, irrespective of host species, have in common the microtubule binding domains and serve the same function. In different models of tauopathy, different human tau isoforms are expressed (either as wild type or harbouring disease mutations), with usually no obvious reason given for the choice of isoform. The results from *Drosophila* models of tauopathy have demonstrated that different tau isoforms (even from the same host species) have differential abilities to cause dysfunction and death. This could be because, as has been demonstrated *in vitro*, they exhibit different interacting partners, different microtubule binding abilities, and different susceptibilities to phosphorylation.

Chen et al., (2007) demonstrated that although the expression of both wild type human tau (2N4R) and *Drosophila* tau leads to the rough eye phenotype in adult flies only about 50% of modifiers identified in enhancer/suppressor screen alter the phenotype of both *Drosophila tau* and human tau in the same way; the remaining modifiers differentially modify the phenotype of either one or the other [42]. This shows that though these two species of tau are functionally similar and may interact with the same proteins in toxic pathways, they do also exhibit significant differences. Kosmidis et al., (2010) have shown that whilst the expression of human wild-type 0N4R tau and 2N4R tau caused a severe ablation of mushroom bodies in adult fly brains, the expression of human wild type 0N3R tau, bovine tau, and *Drosophila* tau have no effect [59]. Interestingly however, despite this lack of effect on the integrity of mushroom bodies, the expression of bovine and *Drosophila* tau leads to learning and memory impairments [10, 51, 65]. This clearly shows a strong isoform specific effect—some isoforms are directly toxic; others cause profound dysfunction rather than overt neurotoxicity.

### 2.6. Tau Phosphorylation—One Size Fits All? 

The insights about the causal role of phosphorylation in the pathogenesis of disease that have emerged from *Drosophila* models are in agreement with those from rodent and other models. However, the findings from *Drosophila* suggest that the pathogenic consequences of tau phosphorylation are dependent upon the site that is phosphorylated. Tau phosphorylation at some sites is associated with neurodegeneration whereas phosphorylation at other sites is associated with neuronal dysfunction in the absence of cell death. Phosphorylation of tau at the PAR kinase/MARK sites (Ser^262^/Ser^356^—the 12E8 site) is associated with toxicity in the *Drosophila* retina [37, 43] and mushroom bodies [59]. However in the study of Nishiumra et al., where they found that the expression of tau not phosphorylatable at the 12E8 site is not toxic, they also noted a reduced phosphorylation at the AT8 and AT100 sites, though significance of this in relation to the toxicity is not clear. They also reported that tau phosphorylation at the PHF-1 site (Ser^396^ Ser^404^) is not dramatically reduced in flies expressing this phospho-mutant tau and hence one may conclude that phosphorylation at this site does not mediate toxicity [43]. This conclusion is further strengthened by the findings of Chatterjee et al., (2009) who also generated that transgenic flies expressing tau are not phosphorylatable at the PAR-1 (12E8) site [37], but unlike Nishimura et al., [43] they coexpressed this with *sgg. *This resulted in the phospho-mutant tau becoming highly phosphorylated at AT8, AT100, and PHF-1, but interestingly, it does not give rise to the rough eye phenotype. Moreover, when Chatterjee et al., expressed another tau phospho-mutant, this time one is not phosphorylatable at GSK-3*β* sites and coexpressed it with PAR-1, they saw a strong 12E8 signal and a severe rough-eye phenotype [37]. Taken together, Chatterjee and Nishimura's findings imply that tau phosphorylated at the PAR-1/MARK sites (like 12E8) causes neurodegeneration, whereas tau phosphorylated at the GSK-3*β* sites (like PHF-1, AT8, AT180, and AT100) causes dysfunction. In support for this we find that in our model, where we do not see any neurodegeneration, manipulations that increase phosphorylation of wild-type human (0N3R) tau at GSK-3*β* sites exacerbate tau neuronal dysfunction phenotypes (such as axonal transport impairments, cytoskeletal disruptions, and locomotor defects) whilst, treatment with GSK-3*β* inhibitors rescues these phenotypes [47, 54]. In a rodent model of tauopathy, Kimura et al., (2007) also report an increased phosphorylation of tau at the PHF-1 site which they associated with dysfunction rather than degeneration [94]. Other tau phosphorylation sites which dissociate tau-mediated toxicity from dysfunction are Ser^238^ and Thr^245^. Kosmidis et al., (2010) reported that the expression of a 2N4R human tau phospho-mutant which cannot be phosphorylated at these sites suppresses the mushroom body ablation that is usually seen following expression of 2N4R tau, but does not abrogate the learning and memory impairment that is also associated with its expression [59]. Their finding that this phospho-mutant tau has elevated AT100 and AT8 immunoreactivity, suggests that phosphorylation at these sites could be associated with dysfunction rather than toxicity. However, since tau has been shown to be phosphorylated at these sites in degenerating neurons [53], one cannot always conclude that occupation of these sites indicates dysfunction and not toxicity. Perhaps the pathogenic effect of any phosphorylation site depends on the other sites occupied on tau at the same time. Furthermore, the phosphorylation profile may change temporally as the disease progresses and dysfunctional neurons begin to degenerate, something that will not be reflected or appreciated when studying the phosphorylation patterns in the end-stage pathology.

## 3. How Do These Insights Pave the Way for Disease-Modifying Therapeutic Intervention?

An unanimous conclusion that one may draw from the findings of *Drosophila* models of tauopathy is that a hyperphosphorylated, soluble species of tau plays a critical role in causing neuronal dysfunction and degeneration. Therefore, there is still a lot of potentials in identifying agents that may reduce tau phosphorylation (some of which have already been tested in *Drosophila*) and research efforts in this area, which are currently ongoing, are likely to yield disease modifying therapeutics. Conversely, the findings from these *Drosophila* models raise doubts as to the usefulness of anti-tau aggregation strategies, at least in the early stages of disease.

With regard to the mechanism by which hyperphosphorylated tau causes toxicity, the findings from numerous *Drosophila* models suggest that tau triggers apoptosis, either by triggering cell cycle activation or inducing oxidative stress. This would suggest that disease modifying interventions to prevent tau toxicity could investigate the usefulness of antiapoptotic or antioxidant strategies. Antioxidant interventions have already shown promise in some *Drosophila* models of tauopathy [56].

The *Drosophila* models of tauopathy that have focused on hyperphosphorylated tau-mediated neuronal dysfunction point to a tau-mediated breakdown of cytoskeletal integrity in the causative mechanism. Disease modifying therapies to counter this could investigate the utility of microtubule stabilising agents, a target that is currently being investigated in the rodent models of tauopathy [108].

The insights that have come forth so far from *Drosophila* about tau turnover would imply that the pharmacological activation of autophagy or the UPR may prove useful for the removal of toxic, truncated, and hyperphosphorylated tau species.

## 4. Conclusion

*Drosophila* models of tauopathy have only been used by tau biologists for the last ten years, and in this time, they have contributed to our understanding of the pathogenesis of tauopathies in a number of ways: (a) they have been pivotal in identifying novel players in the disease process as a result of their amenability to unbiased genetic screens; (b) they have highlighted potential mechanisms of tau-mediated cell death and dysfunction and the significance of the various tau abnormalities in that; (c) they have furthered our understanding of tau turnover in normal and disease states. Stemming from these insights are a number of possibilities for disease-modifying therapeutic interventions, and here too the fruit fly can contribute, by providing the platform for such drug screens in a “whole organism context”. Overall, the ease and experimental tractability of *Drosophila* position it in an important preclinical stage of drug discovery where the exploratory work on disease mechanisms as well as drug testing on the identified pathways can be investigated *in vivo.*

## Figures and Tables

**Table 1:** *Drosophila *genetic techniques used in tauopathy models.

Method	Purpose	How it works	Examples
Gal4/UAS [19, 28]	Allows tissue-specific expression of the gene of interest, in a modular fashion.	One line of flies with Gal4 protein under a promoter expressed in the tissue of interest is crossed with another line harbouring the gene of interest downstream of GAL4-binding UAS DNA sequences and a minimal promotor. In F1 flies with copies of both transgenes, Gal4 will bind to UAS and drive expression, and thus the gene of interest will be expressed only in the tissue of interest.	The majority of *Drosophila* models of tauopathy have used this technique. Tau constructs of different species and isoform, and with a variety of mutations, have been expressed in retina with *GMR*-, *Ey*- or *Sev*-*Gal4* drivers [15, 27, 29–46]; motor neurons with *D42- *or *OK6-GAL4 *[35, 45, 47–49]; sensory neurons with *C161- *or *el6E2-GAL4 *[50]: mushroom body neurons with *c772- *or *C492-GAL4 *[45, 51]; dopaminergic neurons with *Ppk- *or *ddc-GAL4* [38, 52]; cholinergic neurons with Cha-GAL4 [43, 53]; notum with *Eq-GAL4 *[38]; glia with *repo-GAL4 *[25] or panneuronally with *Elav- *or *Appl-GAL4 *[30, 40, 42, 43, 45, 49, 52–63].

Gal80^TS^ (TARGET) [21, 64]	Provides temporal control of the Gal4/UAS system.	Adding another layer to the Gal4/UAS system to drive a gene of interest at the time and place of the experimenter's choosing. Gal80^TS^ is a temperature-dependent repressor of Gal4. Under permissive conditions (18°C), Gal80^TS^ functions normally and blocks Gal4-mediated transactivation of *UAS-transgenes*. At 29°C, Gal80^TS^ is dysfunctional and therefore allows GAL4 to function normally.	Colodner and Feany (2010) used this technique in order to cease expression of human tau in glia after NFTs had already formed (see text) [25]. Papanikolopoulou et al., (2010) used TARGET to study temporal effects of tau phosphorylation in mushroom body neurons [65].

EP lines [66]	A large set of lines of flies with gain or loss of function in numerous genes can be screened for interaction.	Enhancer-promotor-transposable (EP) elements are inserted into the genome at random. They contain GAL4-binding UAS sequences and a promotor. When they land near a gene in the same direction, their activation by Gal4 may promote that gene's transcription in the tissue of interest.	These lines have been screened for modifiers of rough-eye phenotype in flies expressing human tau in the retina [32, 35]. Others have screened the set of lines for modifiers of A-beta [67] or Ataxin-3 [44] toxicity, then coexpressed the hits with tau to identify commonalities.

*UAS-dsRNAi* lines [68, 69]	A comprehensive set of fly lines to knockdown the expression of *Drosophila* genes under GAL4/UAS control.	Interfering double-stranded RNA for a *Drosophila* gene is expressed under UAS. Thus, when crossed with a Gal4 driver, the relevant protein will be knocked down in the tissue of interest. This can also be combined with Gal80^TS^ for temporal control, or the lines used for an enhancer/suppressor screen.	Individual *UAS-RNAi* lines have been used either to knock down tau, or to demonstrate a genetic interaction with tau [46, 52, 57, 70, 71]. A set of 19 lines were used in a secondary screen for modifiers of the tau-induced rough-eye phenotype [27].

MARCM (Mosaic analysis with a repressible cell marker) [72]	Generates a mosaic animal with GFP-marked clonal cells homozygous for an allele of interest, for direct comparison with non-GFP heterozygous or wildtype cells in the same animal.	MARCM relies upon Flp/FRT-mediated homologous recombination in mitotic cells to generate clonal subsets of daughter cells that are either (i) marked with GFP and homozygous for an allele of interest or (ii) heterozygous or wildtype for the allele and unmarked by GFP. Parental cells contain the allele of interest and Gal80 on homologous chromosomes distal to FRT sites as well as *hsFLP, UAS-GFP, *and a cell/tissue-specific Gal4 driver. After recombination, daughter cells which are homozygous for the allele of interest have no Gal80 and will thus express GFP, while nonmarked clones will be wild type or heterozygous.	Nishimura et al., (2004) used this technique to show that *par-1* is required for tau toxicity. Using *elav-Gal4*, they made clones of neurons that were mutant or heterozygous for *par-1* in the presence or absence of human tau overexpression in each combination [43]. This would otherwise have been difficult to test, as *par-1* null flies are not viable.
